# Perturbation Training for Fall-Risk Reduction in Healthy Older Adults: Interference and Generalization to Opposing Novel Perturbations Post Intervention

**DOI:** 10.3389/fspor.2021.697169

**Published:** 2021-08-16

**Authors:** Tanvi Bhatt, Yiru Wang, Shuaijie Wang, Lakshmi Kannan

**Affiliations:** Department of Physical Therapy, College of Applied Health and Sciences, University of Illinois at Chicago, Chicago, IL, United States

**Keywords:** SLIP, TRIP, perturbation, fall, contextual interference

## Abstract

This study examined the effects of perturbation training on the contextual interference and generalization of encountering a novel opposing perturbation. One hundred and sixty-nine community-dwelling healthy older adults (69.6 ± 6.4 years) were randomly assigned to one of the three groups: slip-perturbation training (St, *n* = 67) group received 24 slips, trip-perturbation training (Tt, *n* = 67) group received 24 trips, and control (Ctrl: *n* = 31) group received only non-perturbed walking trials (ClinicalTrials.gov NCT03199729; https://clinicaltrials.gov/ct2/show/NCT03199729). After training, all groups had 30 min of rest and three post-training non-perturbed walking trials, followed by a reslip and a novel trip trial for St, a retrip and a novel slip trial for Tt, and randomized novel slip and trip trials for Ctrl. The margin of stability (MOS), step length, and toe clearance of post-training walking trials were compared among three groups to examine interferences in proactive adjustment. Falls, MOS at the instant of recovery foot touchdown, and hip height of post-training perturbation trials were investigated to detect interferences and generalization in reactive responses. Results indicated that prior adaptation to slip perturbation training, resulting in walking with a greater MOS (more anterior) and a shorter step length (*p* < 0.01) than that of the Ctrl group, would be associated with a greater likelihood to forward balance loss if encountered with a trip. The trip adaptation training mainly induced a higher toe clearance during walking (*p* < 0.01) than the Ctrl group, which could lead to reduced effectiveness of the reactive response when encountered with a novel slip. However, there was no difference in the reactive MOS, limb support, and falls between the control group and the slip and trip training groups on their respective opposing novel perturbation post-training (MOS, limb support, and falls for novel slip: Tt = Ctrl; for the novel trip: St = Ctrl, both *p* > 0.05). Current findings suggested that, although perturbation training results in proactive adjustments that could worsen the reactive response (interference) when exposed to an unexpected opposing perturbation, older adults demonstrated the ability to immediately generalize the training-induced adaptive reactive control to maintain MOS, to preserve limb support control, and to reduce fall risk.

## Introduction

Falls are the leading cause of injury-related deaths among older adults regardless of their physical function and activity level (Rubenstein et al., 1994; Morley, 2002; Spaniolas et al., 2010). Falls often occur without any signs or warnings, even among the healthiest older adults. Large environmental postural disturbances most often lead to slip- or trip-related falls, which comprise 28–53% of outdoor falls (Luukinen et al., 2000; Talbot et al., 2005; Antes et al., 2013). Both types of falls are highly dangerous and can result in fatal injuries such as hip fractures from slips and traumatic brain injuries from trips (Parkkari et al., 1999; Smeesters et al., 2001). The subsequent cost is high after fatal or non-fatal falls (Milat et al., 2011; Towne et al., 2014), and the induced fear of falling leads to activity reduction (Tinetti et al., 1986), diminishing the quality of life of older adults. Due to such vast consequences of falls (social and economic), strengthening the defenses of older adults against falls is imperative.

Efforts toward designing and implementing fall-prevention programs have relied on multifactorial/multicomponent (Hopewell et al., 2018) and single-component interventions (e.g., exercise) (Sherrington et al., 2019). Overall, there is a reduction of 20–30% in the rate of falls by multifactorial/multicomponent interventions and exercises such as Tai Chi (Wu et al., 2010), balance exercises, and functional exercises (Clemson et al., 2012; Arantes et al., 2015). However, it was suggested that the lack of specificity of applying gains from training under a prepared voluntary environment to an unexpected postural disturbance, a scenario that causes slip and trip falls in daily life, might limit the effectiveness of the abovementioned approaches in fall reduction (Grabiner et al., 2014).

Emerging task-specific perturbation-based training paradigms that involve inducing repeated disturbances to the alignment of the center of mass (COM) relative to the base of support (BOS) are known to enhance fall-resisting skills in older adults (Bhatt et al., 2006a,b; Mansfield et al., 2010, 2015; Bhatt et al., 2012; Wang et al., 2012; Pai et al., 2014; Patel and Bhatt, 2015). Perturbations given in a block with pure repetitive slips or trips have been shown to lead to prominent adaptive changes in the performance (Bhatt et al., 2006b; Wang et al., 2012). Critical body disequilibrium in the first perturbation that quickly reduces over a course of repetitive perturbations and is associated with both improved feedforward and feedback control through adaptation (Pai and Bhatt, 2007). Feedback control makes the ongoing reactive adjustments to compensate for motion errors after a perturbation occurs (Wolpert and Ghahramani, 2000), while feedforward control occurs before or in anticipation of a perturbation (Scheidt et al., 2001). Feedforward control makes proactive adjustments to alter the postural control relying on previous experience, and it can also influence the feedback control-related reactive adjustments. Adaptive proactive and reactive stability are achieved by improved control of the relative COM state (i.e., either its position and/or its velocity relative to the BOS; Pai et al., 2003; Pai and Bhatt, 2007). Other than stability, repeated perturbation training is known to significantly improve the control of vertical limb support required to maintain an upright position and minimize hip descent upon a large-scale perturbation. Previous research indicates that such an increase in the post-perturbation reactive limb support is achieved by increased production of the net vertical lower limb joint torque (Pai et al., 2003; Pai and Bhatt, 2007), which, in turn, is influenced by the rate and magnitude of muscle force production. Although adequate studies have reported significant improvements in the reactive balance control and fall reduction following a block of repetitive perturbations generated in the same manner, such a predictive gait alteration induced by predictable block perturbations might obscure the reactive improvements. For example, if participants adopted a high toe clearance before anticipating a trip, it is very likely that they would avoid contacting the tripping obstacle and would make it highly challenging to examine the response of the feedback control (Wang et al., 2019).

It is known that fall mechanisms and corresponding preventive adaptive responses for recovery from slips vs. trips are opposite in nature. For example, a slip or a slip-like perturbation moving the feet/BOS anterior to the COM induces a backward balance loss and associated falls (Bhatt et al., 2006b) and a trip-like perturbation moving the feet/BOS posterior to the COM induces a backward balance loss and associated falls (Wang et al., 2012). Recovery from both thus involves specific directional responses for the control of COM stability. For example, while controlling trunk momentum is crucial for preventing forward falls upon novel trips (Pavol et al., 2001; Wang et al., 2019), a backward compensatory stepping contributes more to slip-induced recoveries (Pai and Bhatt, 2007). Therefore, it is questionable whether such adaptive changes acquired from a highly predictable fixed condition can be transferred to more unexpected conditions with perturbations occurring at random.

To address the above issues, mixed exposure of opposing perturbations (slip and trip) can minimize the anticipation and evaluate the reactive balance response during gait perturbation. A vital form of functional plasticity of the central nervous system (CNS) is its ability to take motor adaptations obtained from one situation and apply them appropriately to different “contexts.” Previous findings have shown the ability of CNS to generalize the adaptive gains in stability and limb support across different environmental contexts (treadmill-slips to over-ground-slips; Lam and Dietz, 2004; Morton and Bastian, 2004a,b; Seidler et al., 2004) or across different tasks (gait-slip to a sit-to-stand slip; Pai et al., 2003; Bhatt and Pai, 2009; Yang et al., 2009, 2013). However, when the contextual difference is large (slip vs. trip), sensorimotor adaptation to a perturbation that requires opposing motor adjustments could, in fact, interfere (negative transfer) with each other, at least in the proactive control of stability. For example, the CNS learns to anteriorly shift the COM position and/or to increase its velocity with feedforward and feedback mechanisms after repeated slip exposure (Pai et al., 2010). Yet, when facing a trip, the CNS must learn to posteriorly shift the COM position and/or to reduce its velocity (Wang et al., 2012). Contextual interference of exposure to slip and trip was proved in young adults. Bhatt et al. (2013) found that proactive adjustments, shown as the anterior shifting of the COM position relative to BOS adapted from prior slip-perturbation training, persisted at the pre-trip instance in a novel trip following the prior slip training. Such proactive adjustments immediately resulted in a greater anterior instability compared with a control group not receiving prior slip training.

It is postulated that the training-induced vulnerability to the opposite perturbation, if existing, could be quickly amended based on the capability of CNS to trigger an adapted reactive control that rapidly enhances post-perturbation stability (improved trunk control and protective stepping) and limb support (improved net vertical joint torque), thus, minimizing the need for an entirely new motor program or immediate improvements in the physical conditions (strength, balance, etc.) of an individual (Morton et al., 2001). The CNS gradually recalibrates and optimizes the stability and limb support gains and its representation of fall risk limits against both forward and backward balance losses. Such a postulation was partially validated in a study conducted in young adults (Bhatt et al., 2013), where such interference seen was, however, mitigated at the post-trip instance of recovery touchdown—a possible generalization of the reactive response resulting in no difference in the vertical limb support and stability values between the training and control groups. Similarly, Okubo et al. (2018) reported that young adults had an improved margin of stability (MOS) when recovering from a trip after exposure to random slip and trip perturbations. There is limited evidence to determine to what extent the interference of the opposing perturbation could affect the proactive and reactive stability control in older adults.

**Figure 1:**
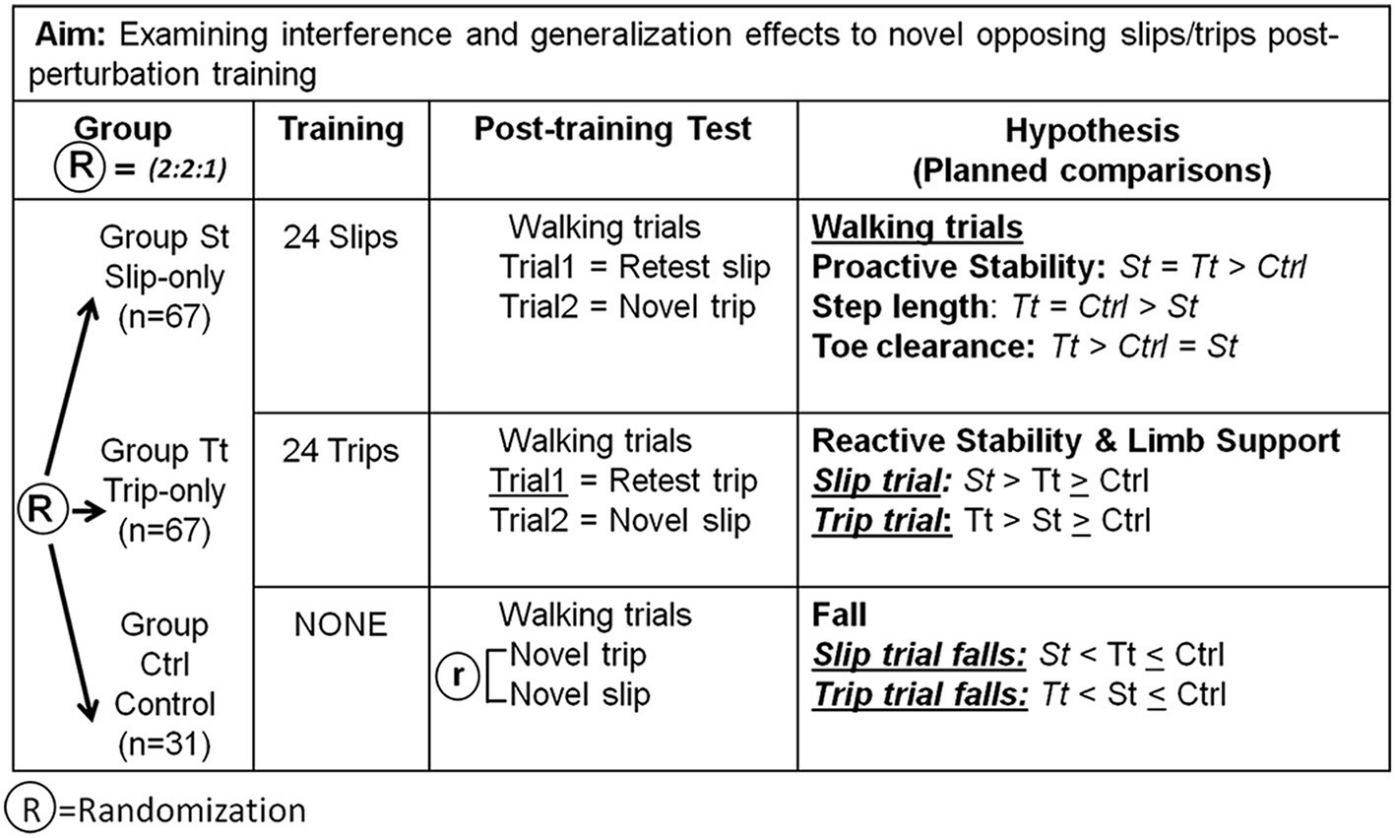
The research design for the hypothesis and the planned comparisons were performed. Post-training walking trials (PW) were compared to examine interferences of training adaptations on the responses to opposing perturbations in the proactive control. Post-training perturbation trials were studied to investigate interferences of training adaptations on the responses to opposing perturbations in the reactive control. R = randomized assignment of subjects among groups.

The aim of this study was thus to determine the effects of perturbation-specific training (slip-only or trip-only) in inducing interference or generalization within proactive (feedforward) and reactive (feedback) mechanisms for the control of stability and limb support, the two likely essential defense elements against falls in older adults. Our prior preliminary results from young adults showed that post-perturbation training, adaptation within proactive control (feedforward), which is involved with the upcoming context prediction, will be prone to a greater interference when exposed to an opposing perturbation (Bhatt et al., 2013). Because we expected that the impact of training-induced improvement in the reactive control of stability and limb support will be higher than that in the proactive control, a proper and effectively trained reactive response can be commonly applicable against falls even under diametrically different precursors. Specifically, we hypothesized that, though perturbation-specific training will induce a negative interference in the proactive control of stability when exposed to the opposing perturbation, it could induce a significant amount of (positive) generalization in the reactive control of stability and limb support, thus, leading to greater gains in these variables and lowering the laboratory-induced falls when exposed to the opposing perturbation compared to that of their controls (Figure 1). Findings from this study can contribute to optimizing the design of an effective perturbation training in older adults.

## Materials and Methods

### Participants

Three hundred and five older adults (>60 years) were initially screened to pass a descriptive questionnaire without the self-reported recent (<6 months) neurological, musculoskeletal, or systematic disorders. Two hundred and forty-one qualified older adults were then screened onsite to pass a cognitive test [>25 on the Folstein Mini-Mental Status Exam (MMSE)] (Mf et al., 1975), a calcaneal ultrasound screening (T score > −2.0) (Thompson et al., 1998), a mobility test [Timed-Up-Go (TUG) score < 13.5 s] (Podsiadlo and Richardson, 1991), and a monofilament foot sensation test (able to detect the Weinstein 5.07 monofilament at all nine locations of both feet; Kumar et al., 1991). One hundred and sixty-five qualified community-dwelling healthy older adults (69.6 ± 6.4 years) were finally included in the study. Participants also received other commonly used clinical measurements and questionnaires, including the Berg Balance Scale (BBS), Activities-specific Balance Confidence (ABC) Scale, a fall history questionnaire, and a 6-min walking test. All participants provided written informed consent, and this study was approved by the Institutional Review Board in the University of Illinois at Chicago.

### Study Design

Qualified participants were randomly assigned following simple randomization procedures to one of the three groups: slip-perturbation training group (St, *n* = 67), trip-perturbation training group (Tt, *n* = 67), and control group (Ctrl: *n* = 31) with a 2:2:1 allocation. This study is the first part of a larger study (ClinicalTrials.gov NCT03199729; https://clinicaltrials.gov/ct2/show/NCT03199729) specifically examining generalization and/or interference effects in older adults when exposed to a directionally opposing perturbation after a slip-only or trip-only training. We had conducted an *a priori* power analysis based on the preliminary data, and because we expected the total rate of slip and trip falls (~30%) in the slip-only and trip-only group to be half of that in the control group (~60%), we needed a larger sample size for slip- and trip-only groups to detect a large effect size between these two groups. The current sample size provided a >80% statistical power to detect a large effect size (=0.5) between the training groups and the control group (slip-only vs. control and trip-only vs. control) and between the two training groups. The randomization option was adopted to maintain sufficient power yet reduce the recruitment burden. A randomization sequence was created using Excel. Group St received 24 repetitive slip perturbations, Group Tt received 24 repetitive trip perturbations, and Group Ctrl received no training but only walking trials. Post-training walking trials were studied to show proactive (feedforward) control. Post-training perturbation trials were studied to indicate reactive (feedback) control (Figure 1).

### Experimental Setup

Slip perturbations were induced by the sudden release of a pair of low-friction, movable platforms on sliding tracks mounted to supporting frames. The two platforms were embedded in the middle of the left and right sides of the 7-m walkway. During slip trials, the movable platform was released when the vertical ground reaction force (GRF) under the perturbed (right) limb exceeded 10% of body weight after the touchdown of the right foot. The left platform was automatically released after the recovery (left) foot landed on it.

**Figure 2:**
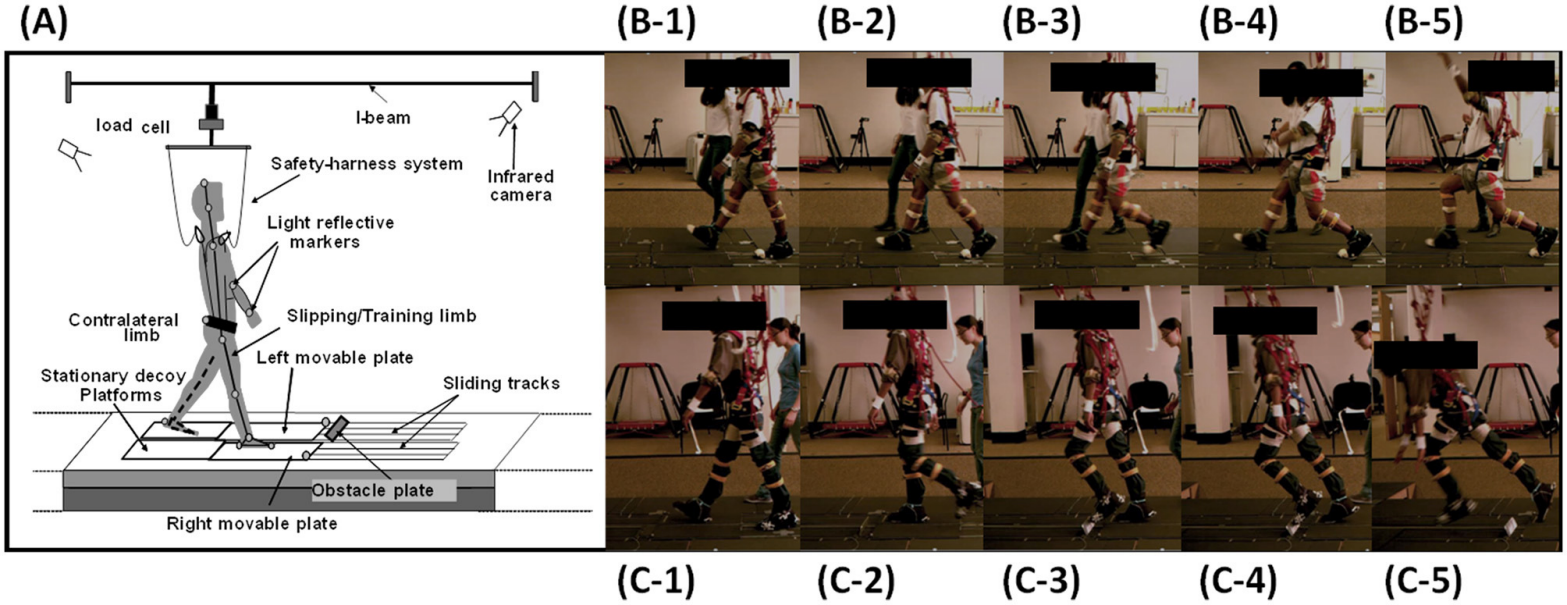
**(A)**. The experimental setup of the over-ground walkway, the overhead harness, and the motion system. **(B-1)** to **(B-5)** Still images indicate the instance of right foot touchdown (RTD) before the slip onset to recovery left foot touchdown (LTD). **(C-1)** to **(C-5)**. Still images indicate the instance of RTD before left foot hitting the obstacle to recovery foot touchdown.

This would guarantee that all slips occurred at the beginning of the double-stance phase (Figures 2A,B). Trip perturbations were induced by an obstacle device (height: 8 cm; width: 27 cm; thickness: 0.5 cm), which was embedded on the left side of the walkway (Figures 2A,C). During trip trials, the trip plate was unlocked after 50 ms of the instant when the vertical GRF under the unperturbed (right) limb exceeded 90% of body weight after its touchdown. Once the trip plate was triggered, it stayed unlocked. This would guarantee that all trips occurred in the late-swing phase. The GRF was detected by the force plates (AMTI, Newton, MA) installed beneath the right platform. During regular walking, both the movable platform and the trip plate were locked by a pair of electromagnets. Participants were protected by a full-body safety harness connected by shock-absorbing ropes to a load cell (Transcell Technology Inc., Buffalo Grove, IL). The load cell was mounted to an overhead trolley on a track over the walkway. The harness enabled participants to walk freely while providing them protection against body impact with the floor. Kinematics from a full-body marker set (30 retro-reflective markers) were recorded by an eight-camera motion capture system (Motion Analysis Corporation, Santa Rosa, CA). Kinetic data were sampled at 120 Hz and synchronized with the force plate and load-cell data, which was collected at 600 Hz.

### Study Protocol

**Figure 3:**
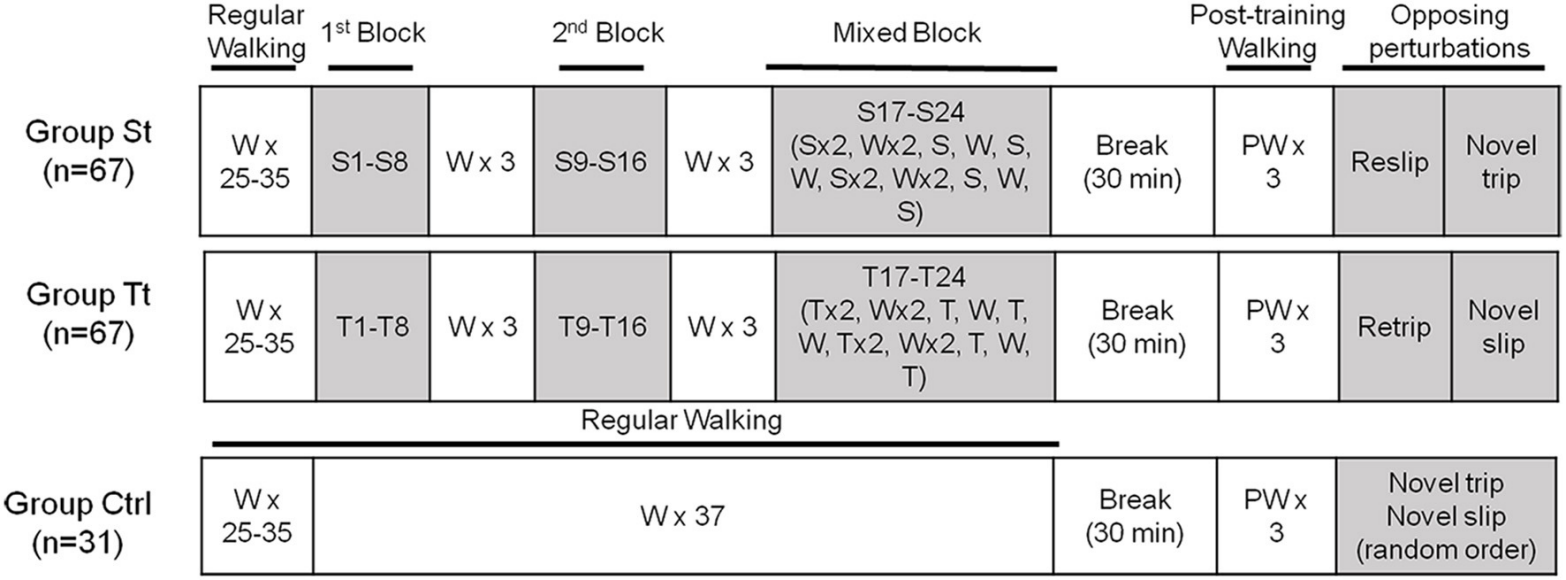
The training protocol used for the study. The slip-training (St) group received 24 repetitive slip perturbations, the trip-training (Tt) group received 24 repetitive trip perturbations, and the control (Ctrl) group received no training but only walking trials (W). Specifically, after 25–35 unperturbed normal walking trials (W) received by all groups, Group St received a block of eight repeated slip trials (S1–S8), followed by three unperturbed trials, another block of eight slip trials (S9–S16), an additional three unperturbed trials, and a final block of 15 mixed trials (including eight slip and seven unperturbed trials) (S17–S24). Group Tt experienced trials in the same design as Group St but trips as perturbation (T). Group Ctrl experienced an additional 37 unperturbed walking trials. After a 30-min break, all groups received three unperturbed post-perturbation walking trials (PW). Then, Group St received a reslip followed by a novel trip, Group Tt received a retrip followed by a novel slip, and Group Ctrl experienced these two perturbations in random order.

All participants experienced 25–35 unperturbed walking trials on a 7-m walkway to become familiar with the laboratory walking environment. Their starting position was adjusted during walking trials to ensure that the upcoming perturbations were consistently induced in the same gait phase for all participants. Specifically, after normal walking trials in the training session, Group St received a block of eight repeated slip trials, followed by three unperturbed trials, another block of eight slip trials, an additional three unperturbed trials, and a final block of fifteen mixed trials (including eight slip and seven unperturbed trials) (Figure 3). Group Tt experienced trials in the same design of Group St but trips as perturbation. Group Ctrl experienced an additional 37 unperturbed walking trials following the familiarization walking session to match the total trials received by the other two groups. After a 30-min break, all groups received three unperturbed post-walking trials. Group St received a reslip followed by a novel trip, Group Tt received a retrip followed by a novel slip, while Group ctrl experienced these two perturbations in a random order. For all three groups, participants were informed that “a slip or trip may or may not occur during your walking” at the beginning of each trial and that, if the perturbation occurred, they should “try to recover and continue walking.”

### Outcome Variables

**Figure 4:**
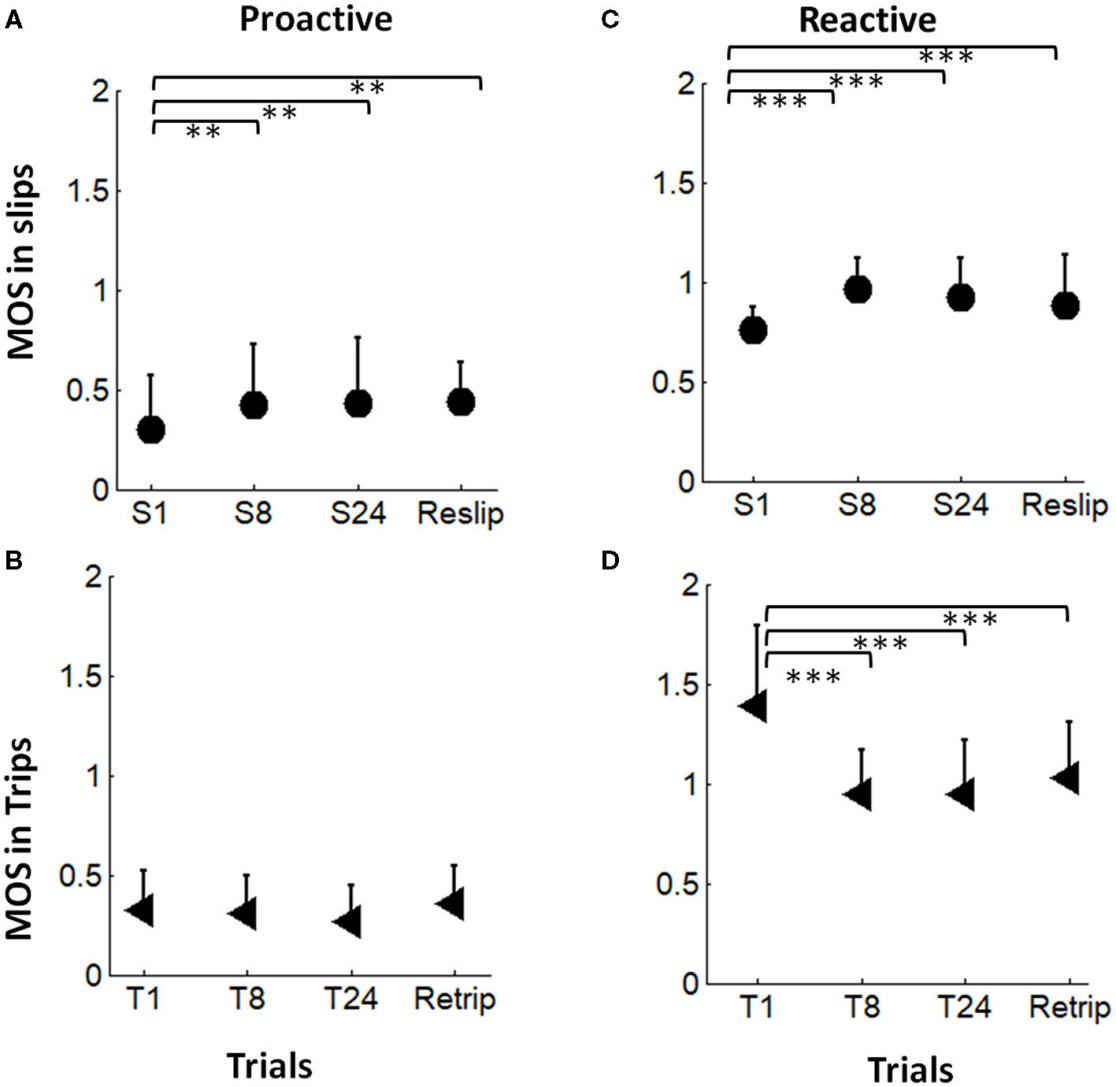
**(A,B)** The proactive adaptation and the short-term (30-min) retention in the margin of stability (MOS) during slip perturbation (indicated by filled circles) and trip perturbation (indicated by filled triangle) trainings. **(C,D)** The reactive adaptation and the short-term (30-min) retention in MOS during slip perturbation (indicated by filled circles) and trip perturbation (indicated by filled triangle) trainings. S1, S8, and S24 indicated the 1st, 8th, and the 24th slips, respectively, during the slip-training session. Reslip indicated the retest slip after a 30-min break. T1, T8, and T24 indicated the 1st, 8th, and 24th trips, respectively, during the trip-training session. Retrip indicated the retest trip after a 30min break. The mean value of MOS and the positive value of standard deviation for each trial are displayed. **p < 0.01; ***p < 0.001.

**Figure 5:**
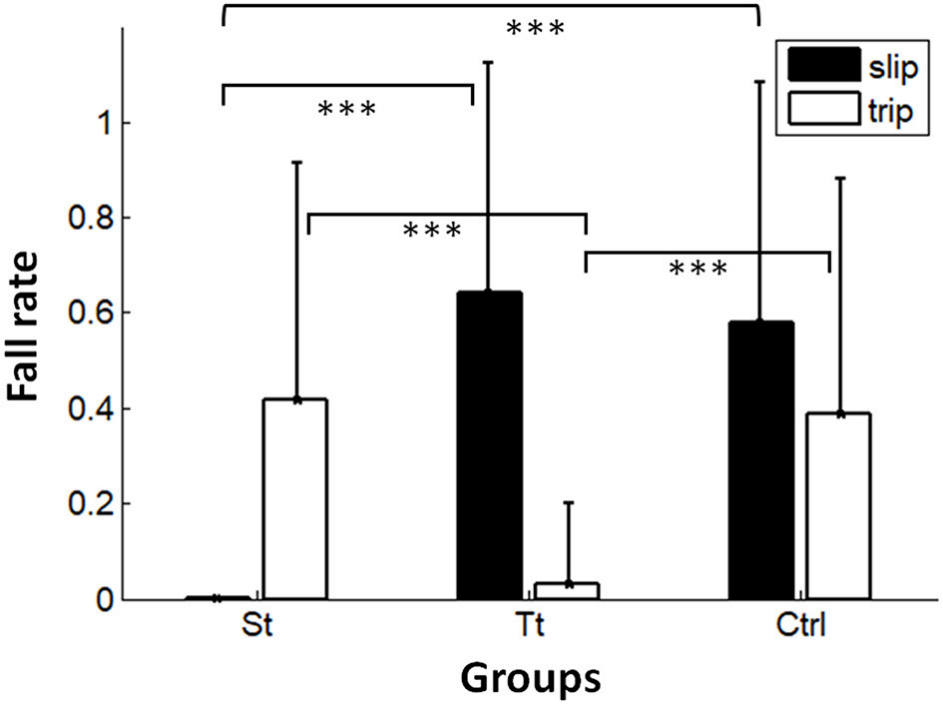
Fall outcomes among the reslip trial in the St group, the novel slip trial in the Tt group, and the novel slip trial in the Ctrl group. Significant differences were shown as the top two lines. Fall outcomes among the retrip trial inTt and the novel trip trial in St and in Ctrl. Significant differences were shown in the middle two lines. ^***^p < 0.001.

Perturbation outcome from a slip or a trip was defined as a fall (Figures 2B-5,C-5) if the load cell detected more than 30% of body weight of the participant after perturbation onset and was further verified using motion videos (Yang and Pai, 2011). If the perturbation outcome did not meet this criterion, it was defined as a recovery. Because both slip and trip were triggered by detecting the right foot touchdown (RTD), the instances of RTD right before a slip (Figures 2B-1) or a trip (Figures 2C-1) onset were chosen to reflect a proactive performance anticipating a perturbation. Following a slip or a trip, the training foot (left foot) quickly touched down for a recovery step; therefore, the instance of the left foot touchdown (LTD) was selected to reflect the reactive response to a perturbation (Figures 2B-4 for the slip and Figures 2C-5 for the trip). All instances were identified from the synchronized vertical GRF and motion analysis data.

Margin of stability was selected to qualify the balance status of an individual, which was calculated as follows (Hof et al., 2005):

$$\begin{array}{l} \text{MOS}=(\text{xCOM}+\frac{\text{vCOM}}{\sqrt{\frac{g}{l}}}-BOS_{pos})/BOS_{len} \end{array}$$

Here, the xCOM indicates the COM position in the anterior–posterior (AP) direction, and vCOM indicates the COM velocity in the AP direction. Body COM kinematics were calculated using a 13-segment rigid-body model with gender-dependent segmental inertial parameters. *g* is the gravitational acceleration and *l* represents the leg length calculated using the markers attached on the greater trochanter of femur. BOS represents the area beneath an individual encircled by the points of contact that the foot or feet of an individual make(s) with the supporting surface, and BOS_pos_ is the posterior edge of BOS, which was calculated using the position of a heel marker. In this study, we normalized the MOS by the length of BOS (BOS_len_), which is the length of BOS in the anteroposterior direction. In this case, the MOS, whose value is >1, indicates that the extrapolated COM exceeds the anterior boundary of BOS, while a negative MOS indicates that the extrapolated COM exceeds the posterior boundary of BOS. A larger MOS indicates better stability against slip perturbation but a greater forward instability against trip perturbation; conversely, a smaller MOS indicates better stability against trip perturbation but a greater backward instability against slip perturbation.

Previous studies indicated that the step length was related to the stability and could affect the risk of slip-induced falls (Espy et al., 2010; Wang et al., 2020). The step length was calculated by subtracting the heel position of the stepping foot from the heel position of the stance foot in the AP direction at RTD. The toe clearance was shown to be highly related to the risk of tripping in older adults (Hamacher et al., 2014). The toe clearance was measured as the maximum vertical distance from the ground to the toe marker in the gait cycle before LTD (from the liftoff of the left foot to its touchdown). Both the step length and the toe clearance were calculated for the post-training walking trial in three groups to indicate the proactive adjustments. The hip height was calculated as the midpoint of the two hip markers. In one gait cycle after perturbation onset (from RTD to LTD), the minimum value of the hip height, calculated when the midpoint of the two hip markers reached the lowest position in the end, was examined during post-training slip and trip trials to indicate the reactive responses. All of these variables were normalized by body height.

Because proactive control quickly improves in the first block through adaptation and remains stable in the subsequent perturbation trials (Bhatt et al., 2006b; Wang et al., 2012), MOS at RTD for S1, S8, and S24 in Group St and for T1, T8, and T24 in Group Tt were analyzed to detect the slip and trip adaptation. MOS at RTD for the reslip and retrip trials were also compared to detect the retention of adaptation. MOS at RTD, step length, and toe clearance in the first post-training walking trials for three groups were compared to examine the proactive adjustment and interference. The first post-training walking trials were chosen to represent the adaptive proactive adjustments because this trial reflects the immediate gait changes used to anticipate a perturbation. MOS at LTD and minimum hip height of reslip and novel trip trials in St, of retrip and novel trip trials in Tt, and of novel slip and trip trials in Ctrl were calculated to examine the reactive interference and generalization.

### Statistical Analysis

One-way ANOVAs were performed to examine any differences in the baseline demographics (age, height, weight, BBS, MMSE, TUG, and ABC) of the participants among the three groups. One-way repeated measures ANOVAs were first performed to examine the adaptive changes and the retention of these changes in MOS (S1, S8, S24, and reslip for St and T1, T8, T24, and retrip for Tt) at RTD and LTD, respectively. Follow-up comparisons were resolved using the paired t-tests between two trials. The Benjamini–Yekutieli procedure is a multiple testing method that controls the false discovery rate under the arbitrary dependence of the *p*-values (Benjamini and Yekutieli, 2001). This procedure was applied to reduce the type I error for multiple comparisons across different groups (corrected *α* = 0.02). A chi-squared test was performed to compare the fall outcomes of a reslip in St, a novel slip in Tt, and a novel slip in Ctrl. A chi-squared test was also conducted to compare the fall outcomes of a retrip in Tt, a novel trip in St, and a novel trip in Ctrl. Furthermore, a chi-squared test was performed between two groups out of the three groups as the *post-hoc* analysis. A fall was coded as 1 and recovery was coded as 0 in the analysis.

A one-way ANOVA was conducted to analyze the training effect (level=3 for Group St, Tt, and Ctrl) on the MOS, step length, and toe clearance at RTD in the post-training walking trials to indicate the proactive adjustments. Independent *t*-tests were used as a *post hoc* test for a two-group comparison (corrected *α* = 0.02). Two-way ANOVA was conducted to analyze the training effect (level=3 for Group St, Tt, and Ctrl), the perturbation effect (level=2 for slip and trip), and the interaction on MOS at LTD, as well as to analyze the minimum hip height in the reslip and novel trip trials of the St group, the retrip and novel slip trials of Tt group, and the novel slip and trip trials of the Ctrl group. Independent *t*-tests were used as a *post-hoc* test for a two-group comparison (corrected *α* = 0.02). Linear regressions were used to examine the relationship between the proactive MOS and the reactive MOS in slip and trip trials. Proactive MOS was input as the independent variable to predict the reactive MOS, which was input as the dependent variable for the slip trials (including the reslip trial in Group St and the novel slip trials in Groups Tt and Ctrl) and the trip trials (including the novel trip trials in Groups St and Ctrl and the retrip trial in Group Tt). All statistical analyses were performed using SPSS 22 (IBM Corp, Armonk, NY).

## Results

### Adaptation and Retention

There was no significant difference in the baseline demographics of the participants (Table 1).

**Table 1:** Baseline demographics and clinical measurements of the participants in the slip-training (St) group, the trip-training (Tt) group, and the control (Ctrl) group.

	**St**	**Tt**	**Ctrl**	***p*-value**
	**(*N* = 67)**	**(*N* = 67)**	**(*N* = 31)**	
Age (yrs)	69.6 ± 6.8	69.9 ± 6.2	68.8 ± 6.4	0.52
Weight (kg)	79.1 ± 18.2	75.7 ± 15.2	78.2 ± 17.3	0.72
Height (m)	1.69 ± 0.1	1.68 ± 0.1	1.65 ± 0.1	0.1
TUG (s)	8.5 ± 1.8	8.2 ± 1.6	8.5 ± 1.5	0.57
BBS	53.5 ± 2.4	53.2 ± 2.9	52.2 ± 2.97	0.11
MMSE	28.6 ± 1.7	28.2 ± 2.3	28 ± 1.8	0.27
ABC	84.4 ± 13.3	85.6 ± 12.3	83.1 ± 15.3	0.7

*The mean and SD of individual variable in each group are provided. P-values of one-way ANOVAs for individual variables among four groups are provided*.

*TUG, Timed-Up-and-Go test; BBS, Berg Balance Scale; MMSE, Mini-Mental Status Exam; ABC, Activities-specific Balance Confidence Scale*.

There were significant differences in the proactive MOS over time in Group St (*F* = 4.85, *p* = 0.003; Figure 4A). MOS rapidly improved in the first eight trials (S8 > S1, *p* = 0.007), and by the end of the slip training, MOS was significantly greater in S24 than in S1 (*p* = 0.005). Training effects remained for 30 min such that reslip had comparable proactive MOS to S24 (*p* > 0.05). There was a trend of reduced proactive MOS from T1 to T24 during trip training (*p* = 0.07) (Figure 4B). There was no significant difference in the proactive MOS between retrip and T24 (*p* > 0.05).

Adaptation of the reactive MOS (at LTD) was observed in both slip training and trip training groups (Figures 4C,D). There were significant differences in the reactive MOS over time for both slip (*F* = 18.5, *p* < 0.001) and trip training (*F* = 28.2, *p* < 0.001) groups. The reactive MOS in S8 and S24 was significantly improved in comparison with that in S1 (*p* < 0.001 for both). Training effects remained for 30 min during which reslip had a comparable reactive MOS to S24 (*p* > 0.05). The reactive MOS in T8 and T24 was significantly lower than that in T1 (*p* < 0.001 for both). Training effects remained for 30 min during which retrip had a comparable reactive MOS to T24 (*p* < 0.001).

### Fall Outcomes

Results of the chi-squared test indicated that fall incidences were significantly different among reslip in the St group, novel slip in the Tt group, and novel slip in the Ctrl group [χ^2^ (2) = 63.0, *p* < 0.001], and the results were significantly different across retrip in the Tt group, novel trip in the St group, and novel trip in the Ctrl group [χ^2^ (2) = 30.1, *p* < 0.001] (Figure 5). For slip-induced falls, the participants in Group St had fewer falls (0%) in reslip than in novel slip in Group Tt (64%) and in novel slip in Group Ctrl (58%) (*p* < 0.001 for both; Figure 5, indicated by filled columns), while no difference was found between Groups Tt and Ctrl (*p* = 0.57). For trip-induced falls, the participants in Group Tt had fewer falls (3%) in the retrip trial than in the novel trip trials in Group St (42%) and in Group Ctrl (39%) (*p* < 0.001 for both; Figure 5, indicated by unfilled columns), while no difference was found between Groups St and Ctrl (*p* = 0.78).

### Interferences in Proactive Adjustments

**Figure 6:**
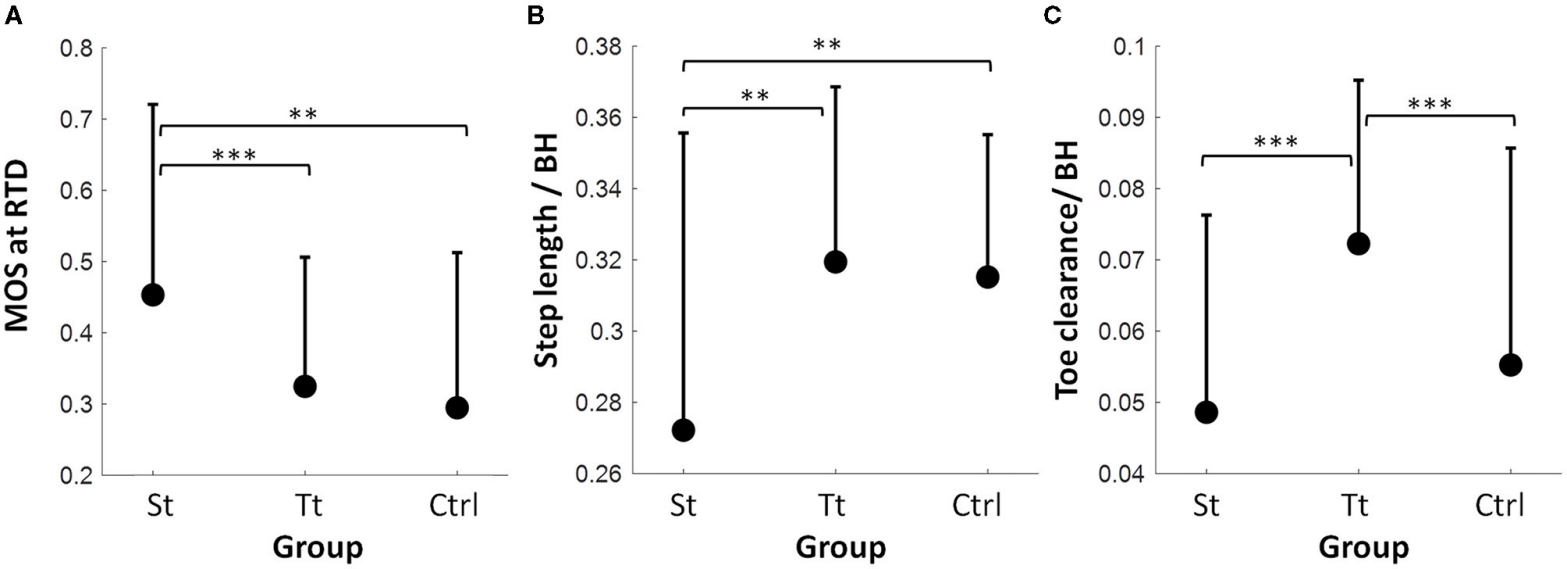
**(A)** Proactive adjustments among PW in the St group, the Tt group, and the Ctrl group in a) the MOS at the RTD, **(B)** the step length normalized by body height (BH), and **(C)** the toe clearance normalized by BH. The mean value of MOS, step length, and toe clearance and the positive value of SD for each variable in each trial are displayed. ^**^p < 0.01; ^***^p < 0.001.

There was a main effect of training on the proactive MOS among post-training walking trials in three groups (*F* = 8.37, *p* < 0.001; Figure 6A). The *post-hoc t*-test showed that the participants in Group St had a significantly larger MOS compared with those in Group Tt (*p* < 0.001) and Group Ctrl (*p* = 0.003), while the MOS was comparable between Groups Tt and Ctrl (*p* = 0.45). There was also a main effect of training on the step length (*F* = 11.2, *p* < 0.001) (Figure 6B) and toe clearance (*F* = 15.6, *p* < 0.001) (Figure 6C). The participants in Group St took a significantly shorter step than those in the other two groups (*p* < 0.01 for both), and no difference in the step length was found between Groups Tt and Ctrl (*p* > 0.05). However, Group Tt had a higher toe clearance compared with other groups (*p* ≤ 0.001 for both), and no difference in the toe clearance was found between Groups St and Ctrl (*p* > 0.05).

### Interferences and Generalization in Reactive Adjustments

**Figure 7:**
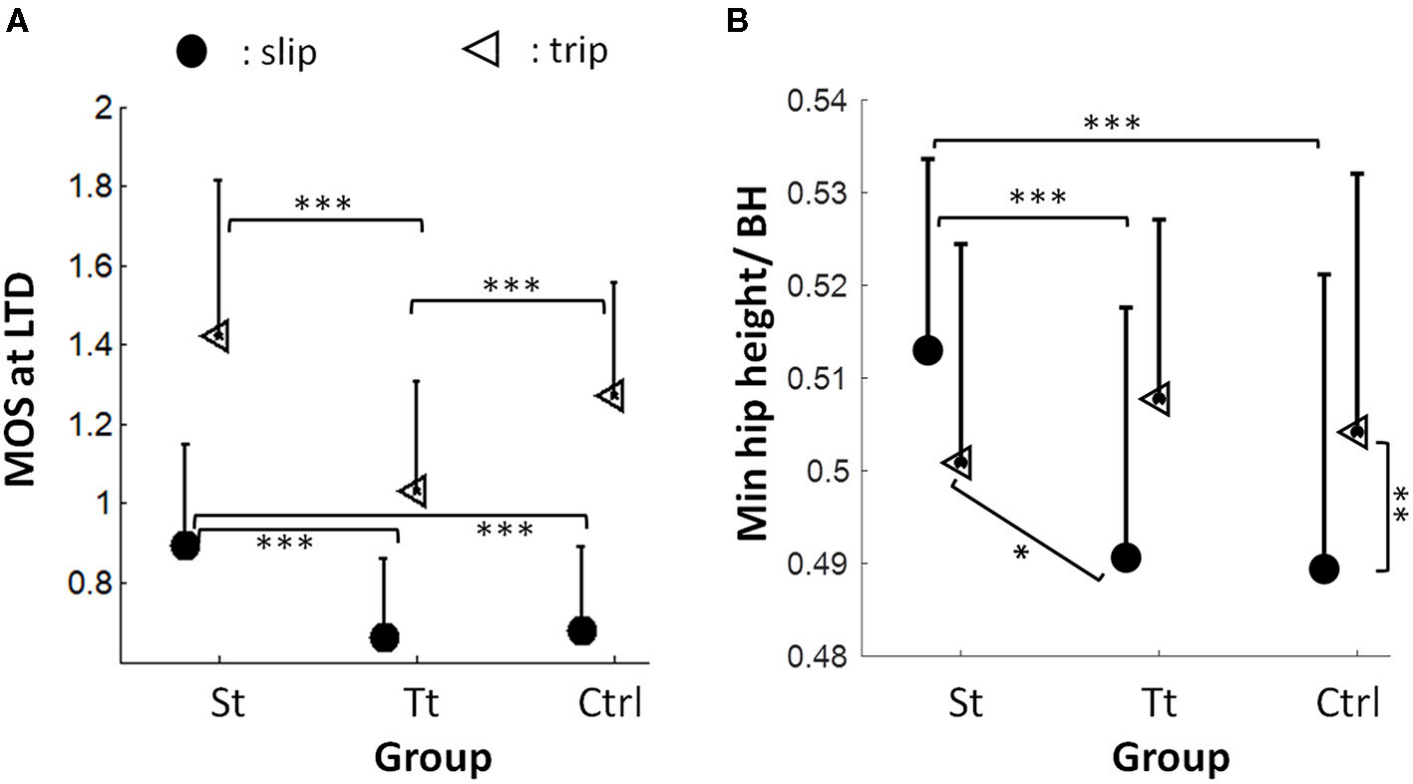
**(A)** Reactive MOS at the recovery LTD among the reslip trial in the St group and the novel slip trials in the Tt group and in the Ctrl group (indicated by filled circles). Significant differences are shown as the bottom two lines. The reactive MOS at the recovery LTD among the retrip trial in the Tt group and the novel trip trials in St and in Ctrl groups (indicated by triangles). Significant differences are shown in the top two lines. **(B)** Limb support (represented by the minimum hip height normalized by BH) among the reslip trial in the St group () and novel slip trials in the Tt group and in the Ctrl group (indicated by filled circles). Significant differences are shown in the top two lines. Limb support among the retrip trial in the Tt group and the novel trip trial in St and in Ctrl groups (indicated by triangles). The vertical line indicates the significant differences between the novel slip and the novel trip of the Ctrl group. The oblique line indicates the significant differences between the novel trip in the St group and the novel slip in the Tt group. The mean value of MOS and limb support and the positive value of the SD for each variable in each trial are displayed. ^*^p < 0.05; ^**^p < 0.01; ^***^p < 0.001.

There was a main effect of the training (*F* = 40.3, *p* < 0.001) and perturbation types (*F* = 230.1, *p* < 0.001), as well as a significant interaction between the training and perturbation types (*F* = 4.8, *p* = 0.009) on the reactive MOS (Figure 7A). Overall, there was a larger reactive MOS in Group St and a larger reactive MOS for the slip perturbations. The *post-hoc t*-test revealed that reslip of Group St had a significantly larger reactive MOS than that in novel slip of Groups Tt and of Ctrl (*p* < 0.001 for both) (Figure 7A, indicated by filled circles), and there were no significant differences in the reactive MOS between novel slips of Groups Tt and Ctrl (*p* > 0.05). The *post-hoc t*-test also indicated that retrip of Group Tt had a significantly smaller reactive MOS than that in novel trip of Group St and of Group Ctrl (*p* < 0.001 for both) (Figure 7A, indicated by triangles), and there were no significant differences in the reactive MOS between the novel trips of Groups Tt and Ctrl (*p* > 0.05). Similarly, there was also a main effect of the training (*F* = 5.08, *p* = 0.007) and perturbation types (*F* = 5.32, *p* = 0.02), as well as a significant interaction between the training and perturbation types on the reactive limb support (hip height) (*F* = 13.74, *p* < 0.001) (Figure 7B). Overall, the hip height was larger in Group St and in trip perturbations. The *post-hoc t*-test indicated that the reslip of Group St had a significantly higher hip height compared with that in novel slips of Groups Tt and Ctrl (*p* < 0.001 for both) (Figure 7B, indicated by filled circles), and there were no significant differences between novel slips of Group Tt and that of Group Ctrl (*p* > 0.05). However, there was no difference in the hip height among retrip in Group Tt, novel trip in Group St, and novel trip in Ctrl (*p* > 0.05 for all). Linear regressions indicated that, for both slip and trip trials, the proactive MOS was a significant predictor (both *p* < 0.001) of the reactive MOS. Specifically, 11.9% (*r*^2^ = 0.119) and 13.9% (*r*^2^ = 0.139) of variances in the reactive MOS were accounted for by the proactive MOS for slip and trip trials, respectively.

## Discussion

Our central hypothesis was that the CNS can still recalibrate its motor strategies based on the commonalities in the reactive control of stability to generalize (positively transfer) the previously learned strategies and to mitigate or overcome any negative interference in the proactive control of stability induced by the opposing perturbation. Specifically, this study supported that post perturbation training, adaptation within proactive control (feedforward), which is involved with the upcoming context prediction, will be prone to a greater interference when exposed to an opposing perturbation. In addition, the current study partially supported that, even with the given negative interference in the proactive control induced by the opposing perturbation training, the training-induced improvement in the reactive control of stability and limb support will be more generalizable than that in the proactive control, as shown by the results that subjects had equal but not inferior reactive stability and post-perturbation limb support gains in comparison to those in the control group who did not receive any opposing perturbations.

This study adopted a design with the first training session of repetitive perturbations of the same type (i.e., all slips or all trips) and a latter part of the mixed exposure of opposing perturbations—one reslip followed by a novel trip for the slip-training group, one retrip followed by a novel slip for the trip-training group, and a randomized novel slip and a novel trip for the control group. Consistent with the previous findings, at the completion of the first training session, subjects demonstrated a trial-to-trial improvement in the reactive control of stability (Figures 4C,D) shown by an increased MOS (more stable against a backward loss of balance) from the 1st slip to the 8th and 24th slips, as well as a decreased MOS (more stable against a forward instability) from the 1st trip to the 8th and 24th trips. Furthermore, the non-significant difference between the retention slip (before the opposing trip) and the 24th slip and the non-significant difference between the retention trip (before the opposing slip) and the 24th trip indicated that such adaptive improvements were able to retain for at least 30 min. Hence, it is reasonable to postulate that, based on the recent perturbation history, the proactive and reactive control of stability would improve or at least remain unchanged given the upcoming perturbation in the same context (Bhatt et al., 2006a; Wang et al., 2019). To meet the demand of sufficient reactive stability against a fall, the CNS has to proactively regulate gait while anticipating an upcoming perturbation in the same context. As shown in our results (Figure 6), proactive stability against a predicted slip was achieved by shortening the step length in the regular gait (step length: St < Tt = Ctrl), and proactive adjustment against a predicted trip was achieved by the increased toe clearance before hitting an obstacle in the regular gait (toe clearance: Tt > St = Ctrl).

From the mechanistic perspective, it is postulated that such an adaptation occurs *via* updating of the internal representation of stability limits based on the immediate and past experiences (Lam and Dietz, 2004; Morton and Bastian, 2004a,b; Seidler et al., 2004). Such an update results in the modification of motor responses (predominantly proactive changes *via* feedforward mechanisms) when the CNS is simultaneously expecting a similar perturbation. When the expected and experienced perturbations match up, it results in an enhanced performance (adapted/learnt response). However, when the CNS experiences a different (in our case, an opposing) perturbation, the proactive adaptive changes could lead to an interference. For example, positive slip adaptations are shown as the shortened step length and a forward-shifted COM position (Bhatt et al., 2006b). Although a smaller step length in gait could bring COM closer to the BOS at the trailing limb lift-off, which, in turn, initiates a better (forward) stability at the slip onset, such a strategy would increase the forward instability for the upcoming trip and could worsen the reactive recovery response (Bhatt et al., 2013). Improved trip adaptations are indicated as a higher toe clearance and a posterior shift of COM (Wang et al., 2019). While sufficient toe clearance can reduce the impact of the trip or completely prevent contact with an obstacle, and a less anterior COM position can establish a stable initial status against forward instability (Wang et al., 2019), such a strategy might increase the predisposition to a backward balance loss and reduce the overall effectiveness of the reactive response upon a slip.

Based on an expected interference resulting from a proactive adaptation, it could be postulated that both St and Tt groups would have more falls, worse reactive stability, and lower vertical limb support than the Ctrl group when they experienced an opposing novel perturbation (novel trip for Group St and novel slip for Group Tt). However, if the generalization of the adaptive improvements through training was demonstrated when experiencing an opposing perturbation, the performance on the novel opposing perturbation would be better than, or at least equal to, that of the control group receiving no prior training.

Our results partially supported such an interference based on the prior expectation for both the St and Tt groups. On the post-training walking trial for the St group, just prior to the 30-min reslip test, we saw that the participants maintained their proactive changes in the slip-training group with a higher pre-slip MOS and a shorter step length than those in the control group and the trip group (who did not get any slip training) (Figures 6A,B). Such proactive changes could have interfered with the reactive response to trips as indicated by a slightly greater forward post-slip/reactive MOS on that trial than that on the novel trip of the Ctrl group (although not significant) (Figure 7A). However, such an interference was probably mitigated by the reactive response demonstrated in the vertical support limb at touchdown of the compensatory step as the proactive MOS only accounted for ~10% variance in the reactive MOS. There was no difference in limb support (Figure 7B) and fall outcomes (Figure 5) on the novel trip trial between St group and the Ctrl group. On the post-training walking trial for the Tt group, immediately before the 30-min retrip test, the toe clearance was higher than that of the Ctrl group and the St group (Figure 6C). However, for trip training, the proactive changes in MOS may not have been retained as robustly as the slip group after 30 min. Thus, there was possibly a lesser proactive interference seen in the trip group, as indicated by a similar proactive MOS between Tt and Ctrl groups (Figure 6A). Subsequently, there was no significant difference in the reactive MOS and limb support on the novel slip between the Tt group and the Ctrl group (Figures 7A,B). However, it must be noted that slips might be more challenging perturbations to recover from than trips, which may help to explain that the limb support on the novel slips for Tt and Ctrl groups (Figure 7, filled circles for Tt and Ctrl groups) was lower than that on the novel trips experienced in St and Ctrl groups (Figure 7, triangles for St and Ctrl groups).

Despite interferences in the proactive control as shown in the Results section, the findings of the non-significant differences in falls, MOS, and hip height in the novel opposing perturbation of the training groups (either St and Tt) in comparison with those in the novel perturbation of the Ctrl group supported our second hypothesis that the reactive control of stability and limb support will be more generalizable than the proactive control, which was consistent with the previous findings. Bhatt et al. (2013) reported that young adults exhibited a lack of difference in the reactive stability after being exposed to opposing perturbations instead of a worsening outcome than their controls without prior interference. This could be explained by a more flexible responding strategy in the feedback control than in the feedforward control. For feedforward adjustment, the CNS relies on prior experience, such as repeated perturbations to recalibrate its internal representation of the fall threshold, and further alters postural response synergies to meet the demand of that specific type of perturbation (Vetter and Wolpert, 2000; Scheidt et al., 2001; Wang et al., 2001; Witney et al., 2001; Davidson and Wolpert, 2003). Hence, the postural responses to an anticipated perturbation are consistent such as a reduced step length when anticipating to a slip or an elevated toe clearance when anticipating to a trip (Bhatt et al., 2006b; Wang et al., 2019). However, the feedback control of gait recovery has more flexibility based on the ongoing COM status. Multiple joint segments together contribute to the global COM state changes and limb support after perturbation onset, and such a multilink mechanism allowed versatile recovery strategies to be applied during gait perturbation (Pijnappels et al., 2004, 2005; Yang and Pai, 2010). For example, after the onset of slip, alteration of stance and swing limbs of the ankle, the knee, and the hip joint led to a change in COM stability (Yang and Pai, 2010), and sufficient knee and hip extensions before training limb liftoff together were major factors preventing a limb collapse (Pai et al., 2006). While after a trip onset, large ankle plantar flexion, knee flexion, and hip extension moments were key to generating the necessary push-off reaction and to restraining the forward angular moment (Pijnappels et al., 2004). Other than lower extremities, a larger peak shoulder flexion post-slip perturbation contributed to a lower fall rate by reducing the trunk extension velocity (Troy et al., 2009). In addition to multiple degrees of freedom adopted in the recovery of gait perturbation and despite proactive interference, muscle responses were rapid enough (usually under 100 ms after a perturbation onset before a recovery step) to allow the online adjustment of reactive control to some extent in both young and older adults (Pijnappels et al., 2005; Pai et al., 2006; Troy et al., 2009).

The findings of this study must be interpreted in light of its limitations. The slips were always introduced during RFT, while the trips were always triggered during left foot swing due to physical constraints in designing the floor for conducting such an experiment; however, in daily life, the slip or trip could occur on either leg. Hence, it was unclear whether such a design would increase or reduce the contextual interference. Moreover, 4% of subjects reported their left leg as the dominant legs, and differences in the dominant leg might contribute to the altered performances in response to perturbations. However, subjects who were left-footed had comparable age, height, and weight, as well as performance in the BBS, TUG, MMSE, and ABC (all *p* > 0.05), in comparison with those who were right-footed (Table 2). Moreover, most of the studies showed no differences between dominant and non-dominant legs in performing dynamic balance tasks in non-athletic adults (Paillard and Noé, 2020). In addition, only healthy older adults were included in the current study, which does not represent more vulnerable older populations who are more likely to fall.

**Table 2:** Demographics and clinical measurements of the participants grouped by leg dominance.

**Dominant leg**	**Right**	**Left**	***p*-value**
	**(*N* = 159)**	**(*N* = 6)**	
Age (yrs)	69.4 ± 6.4	73.7 ± 7.7	0.24
Weight (kg)	77 ± 16	76.2 ± 14.4	0.9
Height (m)	1.67 ± 0.1	1.68 ± 0.1	0.82
TUG (s)	8.4 ± 1.7	8.2 ± 0.9	0.60
BBS	53.1 ± 2.8	53.5 ± 2.7	0.76
MMSE	28.1 ± 2.9	28.5 ± 2	0.67
ABC	84.5 ± 14.7	74 ± 13	0.1

*The mean and SD of individual variables in each group are provided. P-values of the independent t-test analysis for individual variables among two groups are provided*.

In summary, similar to young adults, older adults who received repetitive perturbation training showed the ability to quickly generalize training-induced improvement in the reactive control to overcome negative interference in the proactive control to some extent during novel opposing perturbations. The findings suggest that a future design of perturbation training with mixed opposing conditions may reduce the reliance on feedforward adjustments but enhance the feedback control, which would better prepare older adults to prevent falls in a more complex, highly unpredictable situation that includes realistic environmental fall-risk factors.

## Data Availability Statement

The raw data supporting the conclusions of this article will be made available by the authors, without undue reservation.

## Ethics Statement

The studies involving human participants were reviewed and approved by Institutional Review Board in the University of Illinois at Chicago. The patients/participants provided their written informed consent to participate in this study.

## Author Contributions

TB designed and directed the project. YW, SW, and LK performed the experiments. SW and LK assisted with data analyses. TB, SW, YW, and LK helped with interpretation of results. TB, YW, and SW wrote the manuscript. All authors provided critical feedback and helped shape the research and manuscript.

## Conflict of Interest

The authors declare that the research was conducted in the absence of any commercial or financial relationships that could be construed as a potential conflict of interest.

## Publisher's Note

All claims expressed in this article are solely those of the authors and do not necessarily represent those of their affiliated organizations, or those of the publisher, the editors and the reviewers. Any product that may be evaluated in this article, or claim that may be made by its manufacturer, is not guaranteed or endorsed by the publisher.
